# Study on the Microstructure of a Photonic Crystal Fiber using the Elasto-Optical Effect

**DOI:** 10.1007/s12633-023-02472-w

**Published:** 2023-04-27

**Authors:** Alejandro Sánchez, Alejandro Cortés, Andrés V. Porta, Susana Orozco

**Affiliations:** Department of Physics, Faculty of Sciences, Universidad Nacional Autónoma de México (UNAM), Av. Universidad 3000, Universidad Nacional Autónoma de México C. U. Coyoacán, 04510 Ciudad de México, México

**Keywords:** Photonic crystal, Elasto-optical effect, Optical fiber, Microstructure material

## Abstract

Photonic crystal fibers are characterized by their periodic structure with dimensions in the nanometer to micrometer range, which gives them the potential to be applied in various technical areas. In this work, we study the microstructure of a hexagonal photonic crystal fiber through a macroscopic localized compression test and measurements of relative intensity changes of a transmitted signal in the photonic crystal fiber. Our experimental study was carried out by controlling the orientation of the localized compression respective to the cross-section microstructure of the photonic crystal fiber. To complete the study, we developed a theoretical model based on the elasto-optic effect, and the numerical solution obtained with the model was compared with the experimental results. With both experimental and theoretical results, we obtained a causal correlation between the loss of relative intensity of the signal traveling through the hexagonal photonic crystal fiber and the orientation (respective to the fiber plane) of a localized compression on photonic crystal fiber. In this way, we can explore the cross-section microstructure of a photonic crystal fiber and its orientation in a device with a macroscopic compression test.

## 1 Introduction

Today, optical fibers are the core of long-distance telecommunications, essential for the full implementation of the Internet of Things (IoT) [1, 2] and packet transmission of the Big Data age [3]. On the other hand, optical fibers also enjoy an extensive application in measurement and monitoring techniques [4, 5], in particular, sensors based on photonic crystal fiber have become common in scientific instrumentation and industry [6–9]. Furthermore, the technology for manufacturing optical fibers, and the use of their fundamental characteristics have spread to branches of frontier scientific research, such as gravitation [10, 11], and problems of current interest such as COVID-19 detection techniques [12, 13].

All types of optical fiber are made of structured materials, the periodic structure of photonic crystal fibers defines their transmission properties. This structure has dimensions in the nanometer to micrometer range, so its application is rather complicated in aspects such as the coupling with the emission and detection systems, and the orientation of the structure [14–19], in contrast to some other types of optical fiber such as step-index [20, 21]. In the literature, there are several studies on the deformation of the microstructure of a photonic crystal fiber and the modification of its transmitting properties [22–28]. In some works, this deformation is induced by compressive stresses [22, 23], and in others, the stress is induced by defects deliberately introduced in the structure [24–28]. In the latter, birefringence studies with microstructure photonic crystal fiber designed with different effective refractive indices in two specific directions are of interest [24–26]. In both cases, the cause is the response of

Alejandro Cortés, Andrés V. Porta and Susana Orozco contributed equally to this work.

✉ Alejandro Sánchez
linkale@ciencias.unam.mx

Alejandro Cortés
alexander-2014@ciencias.unam.mx

Andrés V. Porta
avporta@gmail.com

Susana Orozco
sos@ciencias.unam.mx

[1] Department of Physics, Faculty of Sciences, Universidad Nacional Autónoma de México (UNAM), Av. Universidad 3000, Universidad Nacional Autónoma de México C. U. Coyoacán, 04510 Ciudad de México, México

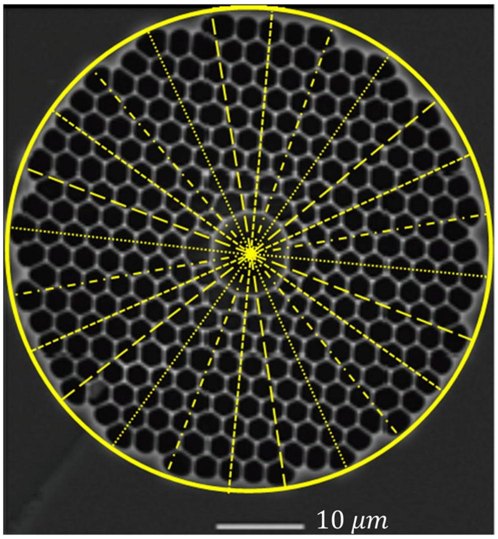

**Fig. 1** The cross-sectional microstructure of the photonic crystal fiber under study. The type lines: dotted, dashed, dot-dash and long-dash, indicate the main radial lines with the rotational symmetry of order six (hexagonal) and whose data are summarized in Table 2

the photonic crystal fiber to variations in one direction of its microstructure produced by mechanical compression.

In this work, we study the microstructure of a hexagonal photonic crystal fiber through a macroscopic localized compression test and measurements of intensity changes of a transmitted signal in the photonic crystal fiber, for which; we designed a device that controls the application direction of a localized perpendicular compression on photonic crystal fiber, respect to the orientation of its cross-section microstructure. The experimental results were compared with a parameterized numerical solution of the problem obtained from a theoretical model based on the elasto-optic effect and previous works by the authors [29–36] and supported by works of other authors in the area [37–44]. The numerical results were adjusted to the experimental results and the parameter numerical values were obtained.

With both experimental and theoretical results, we obtained a causal correlation between the loss of relative intensity of the signal traveling through hexagonal photonic crystal fiber and the orientation (concerning the fiber plane) of a localized compression on photonic crystal fiber. Finally, we discuss how we can explore the microstructure of a photonic crystal fiber and its orientation in a device using a macroscopic localized compression test.

## 2 Experimental Method and Results

Figure 1 shows the cross-sectional structure of the photonic crystal fiber under study. The solid part of the photonic crystal fiber is made of silica and the hollow sections are filled with air, such that the fraction of light in air is greater than 90%. On the other hand, the central operating wavelength of the optical fiber is 1060 nm with an attenuation of 0.00082 dB / cm and, within the range of (700, 1150) nm, the maximum attenuation is 0.00180 dB / cm. Meanwhile, Table 1 shows the geometric characteristics of the optical fiber, and Table 2 contains the data of the main radial lines with order six of rotational symmetry; in the coming sections, four cases relative to these lines are defined, and they will be referred to in the experimental results and the development of the theoretical model.

**Table 1** Geometric characteristics of the photonic crystal fiber

| Characteristic | $\mu$m |
|---|---|
| Core diameter | 10 ± 1 |
| Diameter of region with holes | 50 |
| Cladding diameter | 123 ± 5 |

**Table 2** Characteristics of the main radial lines with rotational symmetry within the cross-sectional microstructure of the photonic crystal fiber

| Type line | Number of radial lines | Average number of holes crossing |
|---|---|---|
| Dotted | 6 | 8 |
| Dashed | 6 | 5 |
| Dot-Dash | 6 | 3 |
| Long-dash | 6 | 3 |

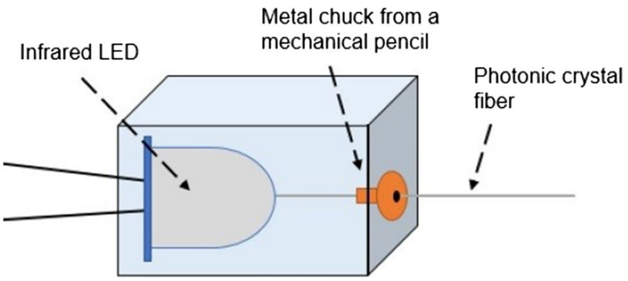

**Fig. 2** Fiber to the LED coupling

A photonic crystal fiber with length 5 cm was used to transmit a constant intensity optical signal generated with an infrared LED IR33CC with a peak wavelength of 940 nm and a spectrum width of 40 nm. To detect the emitter signal, a phototransistor whose detection peak is 940 nm was used. Figure 2 describes the coupling between the LED and the optical fiber; an equivalent coupling was used between the phototransistor and the fiber.

Between the emitter and receiver circuits, the photonic crystal fiber was placed with a fixed and arbitrary orientation. In the experiment, the orientation of the cross-sectional structure of the photonic crystal fiber relative to the connectors between the emitter and receiver circuits remained fixed.

To control the direction of localized compression on the photonic crystal fiber, we designed a device that allows the application of localized and perpendicular compression on the photonic crystal fiber using a press. Figure 3 shows the angle of the press with respect to the fiber plane. The angle variation was controlled using the device.

Figure 4 shows the parts of the press that consists of a steel rod, a spring, and a micrometer screw. The compression of the spring is engaged to the advance of the screw in the press; to quantify the force, the parameters of the press components were calibrated through compression essays in the laboratory. The advance of the screw indicates the compression of the spring, and therefore, the force that is applied on the fiber, which is distributed over the contact area between the fiber and the rod. Therefore, the independent variables of our experiment are the force applied in the localized compression process, and the angle concerning the fiber plane, in which the press is placed.

For each angle of the press, the photonic crystal fiber with coating and jacket was compressed quasi-statically, and the relative intensity of the transmitted signal was measured, that is the ratio between the intensity of the signal with the fiber under localized compression and the intensity of the signal without compression on fiber. Figure 5 shows the variation of relative intensity as a function of the applied force, in the $(10°, 120°)$ range. In all cases, for force values greater than 400 N, the relative intensity decays exponentially.

The relative intensity variation at different angular positions of the press is hard to observe in Fig. 5. To reveal the correlation between the direction of localized compression and the relative intensity change of the transmitted signal in the fiber, graphs of relative intensity against the angular position of the press (with respect to the fiber plane, see Fig. 3) were made with these data, showing their uncertainty bars for each one, and after interpolating, it was observed that each curve associated with a compression force value had a pattern of local maxima and minima, see Fig. 6. The uncertainty bars allow us to discern between the curves according to the resolution of our measurement instruments, and the results mentioned below take this into account.

In each curve, it is observed that for the first, third, fifth, and seventh local maxima, from left to right, there is a mean angular separation of 0.52 ($\approx \pi/6$), and a mean angular separation of $0.482 \pm 0.176$ ($\approx \pi/6$) exists between the first, third, fifth, and seventh local minima, from left to right, this result is in agreement with the symmetry of the photonic crystal.

The angular separation between the local maxima and minima of each of the curves in Fig. 6 are correlated with the hexagonal symmetry of the photonic crystal fiber cross-section and the radial direction of perpendicular and local compression on the photonic crystal fiber, see Fig. 1, because the maxima and minima pattern is the same for all force values between 415 N (on the top) and 996 N (on the bottom), for higher compression forces, more considerable changes in the relative intensity are obtained.

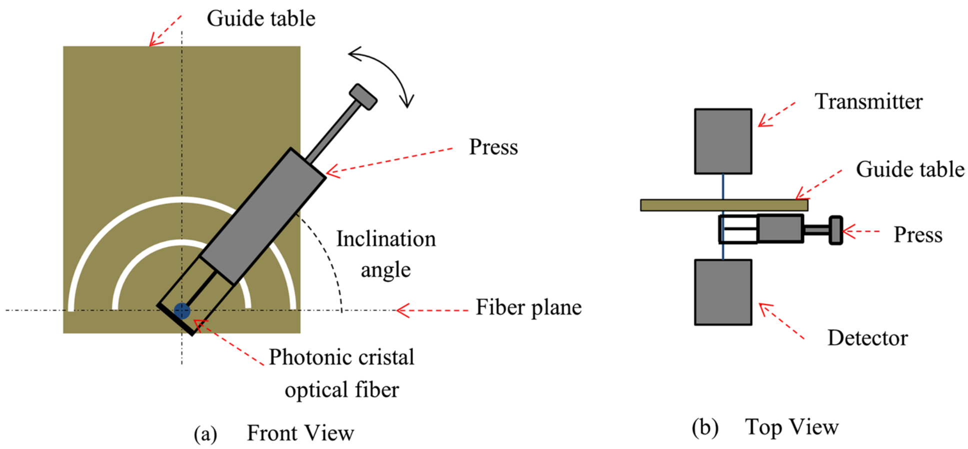

**Fig. 3** Schematic of the experimental device

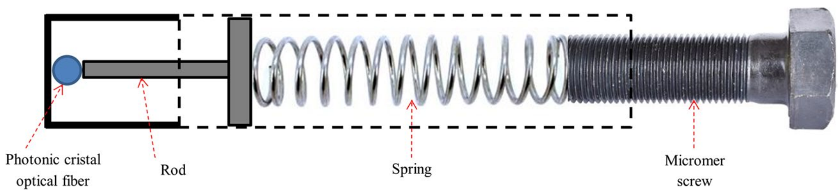

**Fig. 4** Schematic of the press

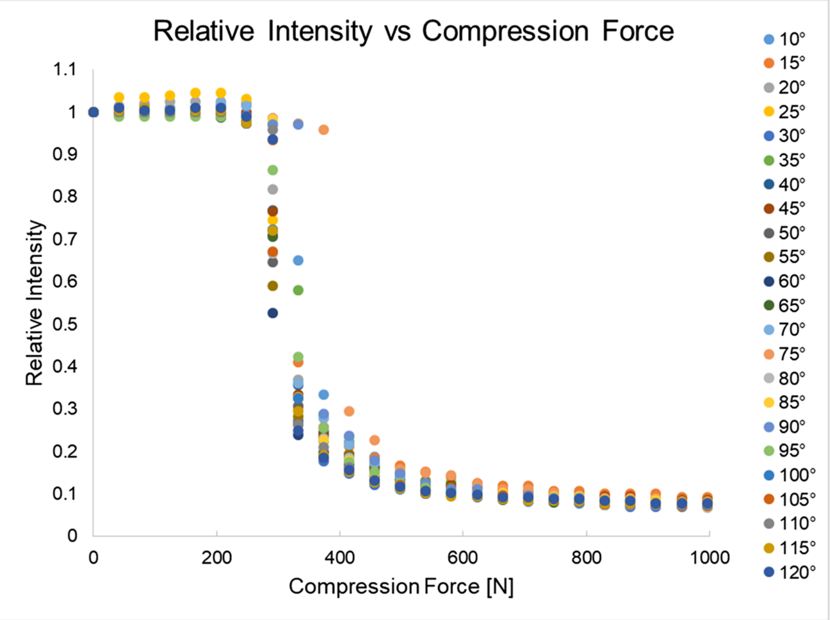

**Fig. 5** Relative intensity vs compression force for different angular positions of the press

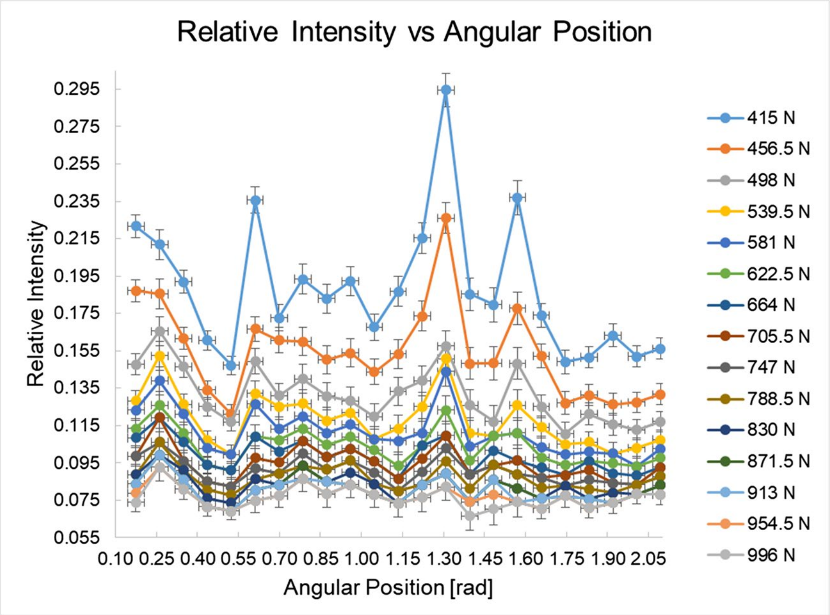

**Fig. 6** Relative intensity vs angular position of the press with respect to the plane of the optical fiber

Finally, it is experimentally clarified that the relative intensity has a periodical behavior with the angular position of the press, and the data presented in Fig. 5 corresponding to the same angular position of the press draw a curves family with the same qualitative behavior with the magnitude of the compression force. We affirm that the behavior of the data for compression force values greater than 400 N corresponds to exponential decay, because the data were fitted in logarithmic graphs with straight lines and, on average, a coefficient of determination $R^2 = 0.9391$ was obtained. Six of these adjustments are shown in Fig. 7. The continuous line corresponds to the linear fit and, the points are the logarithms of the experimental data, with their respective uncertainty interval.

## 3 Theoretical Model and Numerical Solution

To complete our study, we developed a theoretical model based on previous work by the authors and the characteristics of the photonic crystal fiber under investigation, as explained below.

As was previously calculated in the reference [29] from the eikonal equation, the ratio of the intensity of an optical

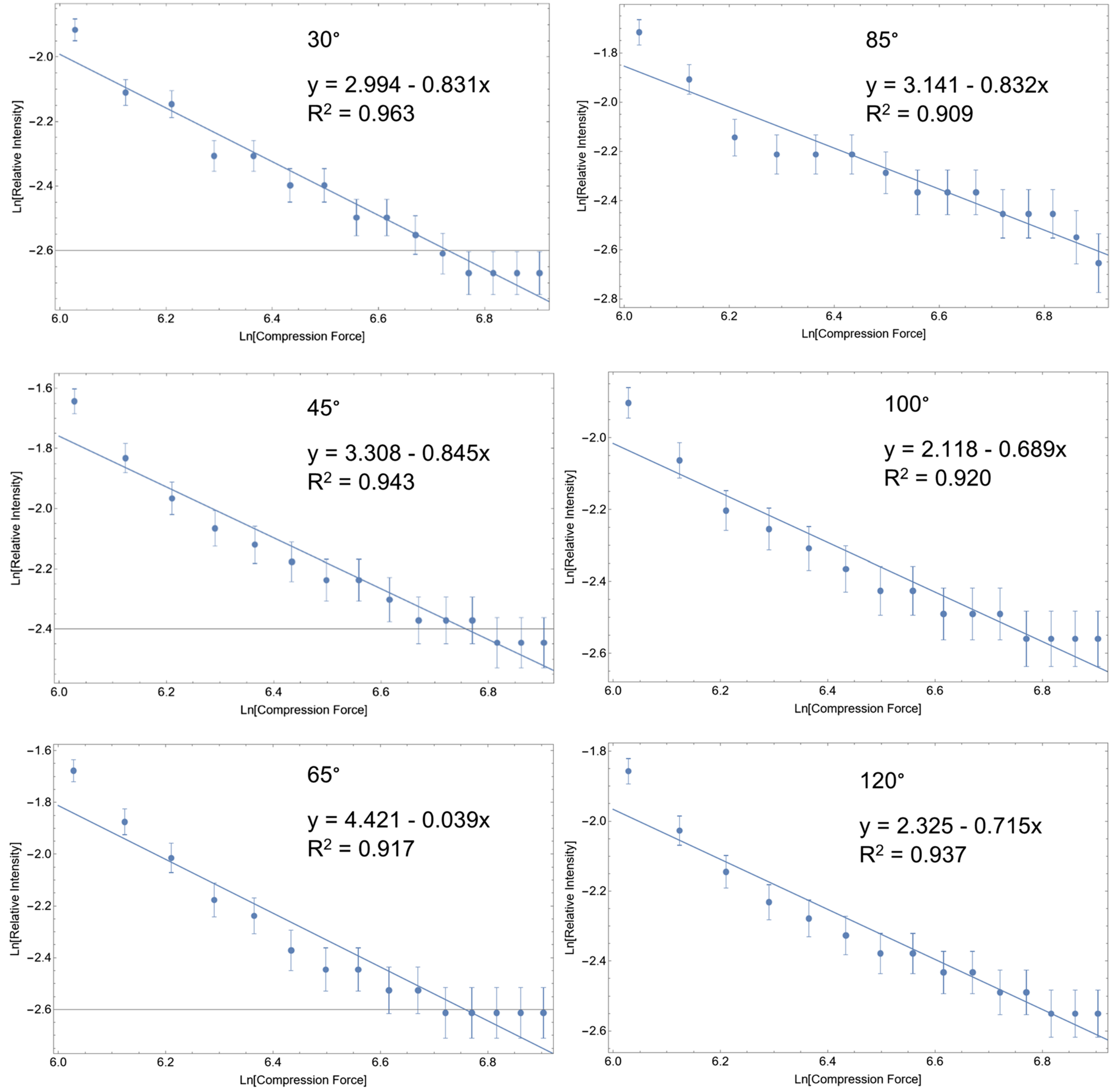

**Fig. 7** Logarithmic graph of experimental data for relative intensity vs compression. The points with the uncertainty interval correspond to experimental data and the solid lines correspond to the linear fits. On the left, from top to bottom, the angular positions of the press are: $30°, 45°, 65°$; and on the right, from top to bottom the angular positions are: $85°, 100°, 120°$

fiber under pressure $I(\theta, F)$ to the original intensity $I_o$ is given by:

$$\frac{I(\theta, F)}{I_0} = \frac{n(\theta, F)}{n_o} e^{\left[-\iint_{r_0,0}^{r,2\pi} \frac{\nabla^2 S(r,\theta)}{n(\theta,F)} dr d\theta\right]},$$

where $n_0$, is the refractive index of the fiber without compression and $n(\theta, F)$ is the refractive index of the fiber under the action of an external force $F$; and $S(r, \theta)$ is the eikonal function, which depends on the magnitude of the position vector $r$ and the angle $\theta$. Once the integrals are evaluated, with respect to the variables $r$, and $\theta$, the exponential function must be decreasing function of the force $A(F)$:

$$e^{\left[-\iint_{r_0,0}^{r,2\pi} \frac{\nabla^2 S(r,\theta)}{n(\theta,F)} dr d\theta\right]} = A(F), \tag{1}$$

which satisfies that when $F = 0$, $A \rightarrow 1$, and $\frac{I(\theta,0)}{I_0} = 1$, therefore,

$$\frac{I(\theta,F)}{I_o}=\frac{n(\theta,F)}{n_0}A(F) \tag{2}$$

We consider that the localized compression on the fiber is spread in a Gaussian distribution around the point of application. In analogy to our study on localized compression on step-index fibers [29].

In this previous work, the loss of intensity due to localized pressure was theoretically calculated using a model based on the elasto-optical effect and compared with experimental data. The intensity that agrees with the experimental results depends on the refractive index. The refractive index, in turn, is a gaussian function of the position and the pressure. This functional dependence was calculated considering the optical anisotropy that arises from the elasto-optical effect. In a medium with a homogeneous cross-section, the anisotropy is independent of the radial direction. Nevertheless, in this study the cross-section symmetry of the photonic crystal fiber is hexagonal, the application of the compression must cause a different effect according to the radial line in which the compression is applied, see Fig. 1 and Table 2, and must be the same for the same type of radial lines. That is, the response of the photonic crystal fiber to localized compression must be anisotropic with hexagonal symmetry. As shown by the experimental results of this work.

To calculate $\frac{n(\theta,F)}{n_o}$ when a force is applied in direction $\theta$, we consider that the compression is distributed in a gaussian shape around the application point, the width of the distribution spans a set of radial lines with a different number of holes.

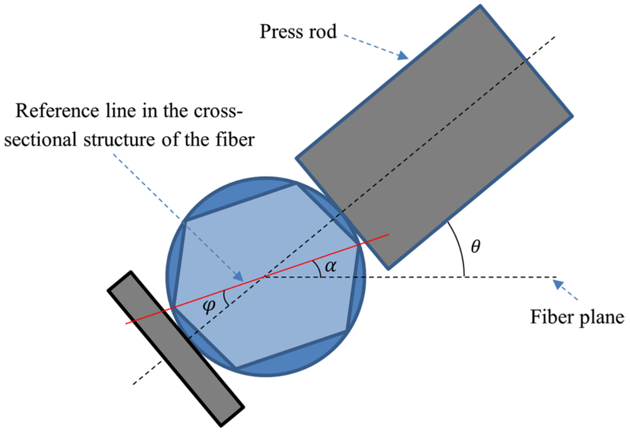

**Fig. 8** Angle $\theta$ of the press rod with respect to the fiber plane, angle $\phi$ of the press rod with respect to a reference line in the the cross-sectional structure of the fiber, and the angle $\alpha$ of cross-sectional structure of the fiber with respect to the fiber plane, which is fixed in the experiment but is unknown

The first term of the summation with $i=0$, where $C_0=0$ is introduced to guarantee that, when $F=0$, $\frac{n(\theta,0)}{n_0}=1$. The parameters in Eq. (3) will be defined later.

Additionally, the transmitted signal along the photonic crystal fiber is also a function of the angle $\phi$ of the press rod relative to a reference line in the cross-sectional structure of the fiber, see Fig. 8. Since the angle $\alpha$ of the cross-sectional structure of the fiber with respect to the fiber plane is unknown, then the angle $\phi$

$$\frac{n(\theta,F)}{n_0}=\sum_{i=0}^{4}e^{-\left(\frac{c_i}{73}\left[senk_i\theta-B_i\right]^2\right)}=\sum_{i=0}^{4}\frac{1}{e^{\frac{C_i}{73}\left[senk_i\theta-B_i\right]^2}}=\sum_{i=1}^{4}\frac{1}{1+e^{\frac{c_i}{73}\left[senk_i\theta-B_i\right]^2}} \tag{3}$$

is also unknown. Remember that in the experiment only the angle $\theta$ of the press rod with respect to the plane of the fiber is measured, see Fig. 8. Theoretically, we propose four main cases that probably can occur in any of the compression tests (different values of the angle $\theta$). Each one of these four main cases is associated with the radial lines in Fig. 1 and the data in Table 2, and are quantified by the parameters $k_i$, $B_i$, and $C_i$ (with $i=1,2,3,4$); $k_i$ is associated with the rotational symmetry of cross-sectional of the fiber, $B_i$ is the width of the Gaussian distribution around a radial direction, and $C_i$ is related to the strain ratio of the photonic crystal fiber and a silica fiber without holes due to compression the dielectric properties of silica and air change, as was explained in the references [29, 32–41]. This parameter considers the effect on the deformation of the photonic crystal fiber by the number of holes in different radial directions.

From Eqs. (2) and (3), we propose that the change in relative intensity of the optical signal traveling through the photonic crystal fiber as a function of the angle $\theta$ of the press rod and the force magnitude $F$ is given by:

$$\frac{I}{I_0}(\theta,F)=A(F)\sum_{i=1}^{4}\frac{1}{1+\exp\left\{\left(\frac{C_i}{73}\right)\left[\sin\left(k_i\theta\right)-B_i\right]^2\right\}}, \tag{4}$$

where $I/I_0$ is the relative intensity, $\theta$ is the angular position of the press relative to the fiber plane, $k_i$, $B_i$ and $C_i$ are the parameters defined above, 73 is the Young's modulus of silica in GPa such that $73/C_i$ is the ratio of the photonic crystal fiber strain to the strain of the silica fiber without holes $\epsilon^{(h)}/\epsilon^{(s)}$:

$$\frac{\epsilon^{(h)}}{\epsilon^{(s)}}=\frac{Y^{(s)}}{\frac{Y^{(h)}\sigma^{(s)}}{\sigma^{(h)}}}, \tag{5}$$

where superscripts $(h)$ and $(s)$ indicate photonic crystal fiber and silica fiber without holes.

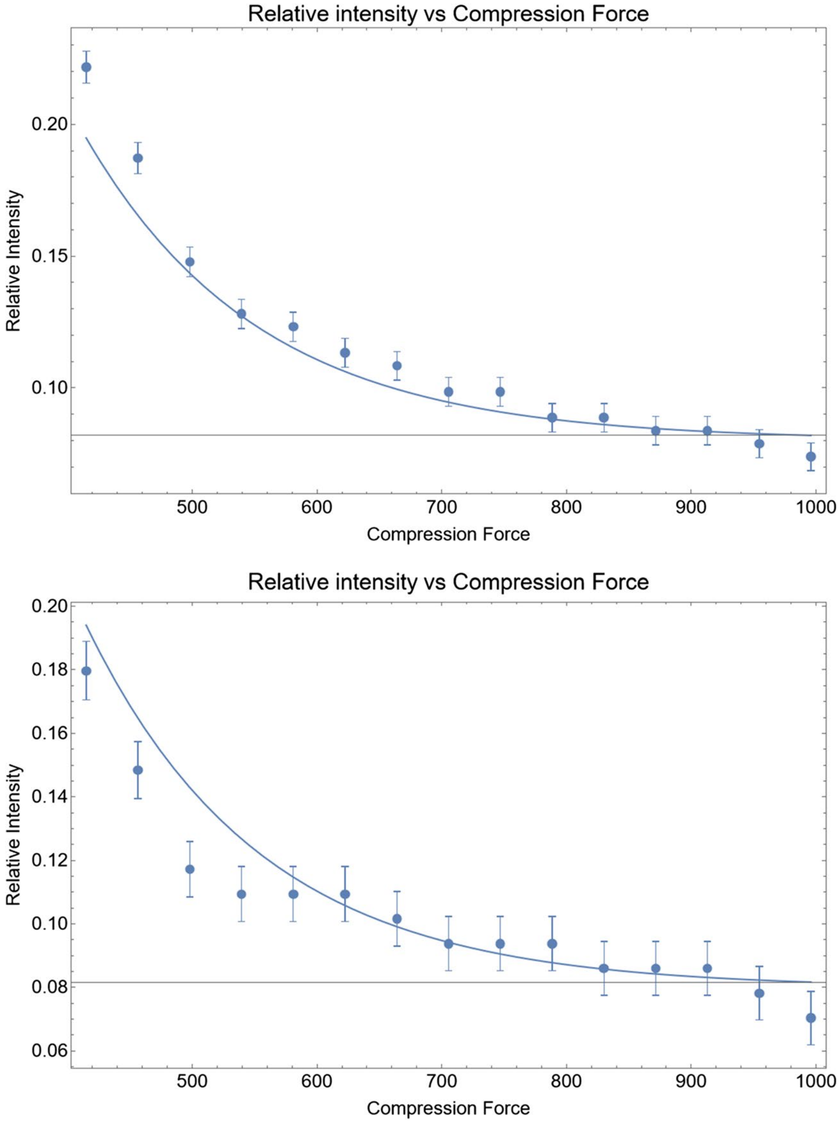

**Fig. 9** The exponential decay curves were calculated with Eq. (4), the points with uncertain bars are experimental data for angular positions values: 10° (top) and 85° (bottom)

In Eq. 4, for each one of the four main cases ($i = 1, 2, 3, 4$), the numerical value of the parameter $k_i$ is defined by a statistical frequency of compression tests, and this is associated with a particular rotational symmetry, the value of the parameter $B_i$ correspond to the width of the Gaussian distribution, and the value of $C_i$ is associated with the number of holes present in the compression directions and is determined by a fit with the experimental data. The definition of each one of the four main cases is detailed below.

The first main case corresponds to the rod centered on one of the 24 angular sections of the cross-sectional structure shown in Fig. 1. Therefore, this case contributes with a frequency $k_1 = 24$ for the total of compression tests. The relative amplitude of the angular distribution is the smallest of all the main cases and was assigned $B_1 = 1$. With this main case, all the subsequent values of $B_i$ were normalized. The average number of holes aligned to the center line of each of the 24 angular sections is three, and the fit with the experimental data gave $C_1 = 12$GPa.

When the axis of the rod is centered on either dot-dash lines or long-dash lines, we define the second main case. There is a contribution of two rotational symmetries of order six; see Fig. 1 and Table 2. This main case contributes with a frequency $k_2 = 12$ for the total compression tests. The relative amplitude of the Gaussian distribution of the compression centered on any of these radial lines is larger than in the previous main case and was assigned $B_2 = 4$. On average, there are only three holes aligned to each of these radii, and the fit with the experimental data gave $C_2 = 15$GPa.

The third main case corresponds to a compression where the axis of the rod is centered on one of the dashed lines and they have associated a symmetry rotational of order six. Therefore, this main case has a contribution with frequency $k_3 = 6$ for the total of compression tests with different angular positions $\theta$ of the press concerning the fiber plane. The relative angular amplitude of the Gaussian distribution of the compression is greater than in the previous main case and was assigned a value $B_3 = 8$. Each radial line cross five holes in the cross-sectional structure, see Fig. 1 and Table 2, and the fit with the experimental data gave $C_3 = 8\text{GPa}$, because the number of holes aligned with the radial direction is more than in the previous main case.

Finally, the fourth main case corresponds to compression with the rod centered on one of the dotted lines and they have rotational symmetry of order six. Therefore, this case has a contribution with frequency $k_4 = 6$, for the total of compression tests with different angular positions $\theta$ of the press with respect to the fiber plane. The relative angular amplitude of the Gaussian distribution of the compression, centered on any of these radial lines, is equivalent to the previous case and was assigned $B_4 = 8$. These radial lines cross eight holes of the cross-section structure, see Fig. 1 and Table 2, and the fit with the experimental data gave $C_4 = 8$ GPa because the photonic crystal fiber presents less resistance to compression in the radial directions with a greater number of aligned holes.

The numerical values of the parameter $A(F)$ were determined after adjusting the numerical solution with the experimental data of relative intensity against the angular position, for each value of the compression force. The parameter $A(F)$ only depends on the compression force. To verify this peculiarity of the theoretical model, we compared the numerical solution of the relative intensity against compression force with the experimental data for forces greater than 400 N and with the determined values of parameter $A(F)$, whose fit gives the function $A(F) = 0.10781 + 0.89545e^{[-0.00317F]}$, with $R^2 = 0.9919$. This equation satisfies the condition, that $A(0) = 1$. For these values of the parameter the relative intensity was calculated as a function of the force using Eq. (4). Figure 9 shows the settings for the angular position values 10° and 85° for forces greater than 400N, the relative intensity decays exponentially as can it observed in the curves of Fig. 9, in this figure also the experimental points are shown, with their respective uncertainty bars.

Finally, after adjusting all numerical results of the model with the experimental data. Numerical values of the parameters of the theoretical model were fixed, in Fig. 10, all numerical results are shown as graphs of relative intensity against the angular position of the press for all values of the compression force used in the experiment.

The numerical results of the model have a root-mean-square error of 0.0002 with the experimental data for the relative intensity, and the same qualitative behavior.

## 4 Discussion

The numerical results of the theoretical model indicate that, for all force magnitudes, the angular differences between the maxima and minima correspond to the values of the experimental results and the symmetry of the fiber cross-section. Furthermore, the theoretical model explains why they are maxima and minima patterns: Since a larger number of aligned holes in a radial direction of the photonic crystal fiber cross-section implies a lower resistance of the fiber to local compression, and due to the elasto-optical effect, the loss of intensity is more significant. Moreover, the numerical results show that there are maxima and minima patterns separated by the rotational symmetry of order three, whose origin is the superposition of the main cases in the theoretical model. These patterns are also present in the experimental results: second and sixth local maxima and minima, see

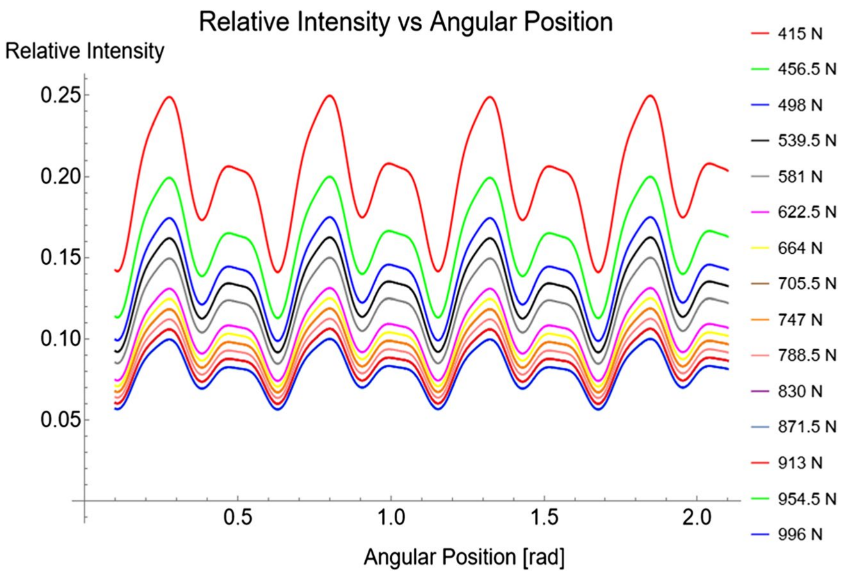

**Fig. 10** Numerical results of the model for the change in relative intensity $I/I_0$ against the angular position $\theta$ for a fixed compression force value

Figs. 6 and 10. The points for 10° with forces of 425 N and 456 N, in Fig. 6 are atypical; these differences larger than the others made increased the RMS value. This error must be compared with the uncertainty of the measured relative intensities (0.0001), therefore the RMS is two times the uncertainty of each experimental data. In the other points of Figs. 6 and 10, there is a qualitative agreement, including the other data for $\theta = 10°$, with forces greater than 498 N.

Both experimental and theoretical results enable us to distinguish the geometry of the photonic crystal fiber and define its angular orientation through a macroscopic compression test.

## 5 Conclusion

Our experiment consisted of a constant intensity signal transmitted in a hexagonal photonic crystal fiber whose cross-sectional microstructure remained fixed in the experimental device. The photonic crystal fiber was subjected to localized perpendicular compressions, and the compressions were angularly oriented concerning the cross-section microstructure of the fiber using a press positioned with respect to the plane of the fiber in the device. We found that the change in the relative intensity of the signal against the angular position of the press, for a value of localized compression force, presents a pattern of local maxima and minima, whose amplitudes depend on the direction of compression concerning the microstructure of the cross-sectional of the hexagonal photonic crystal fiber. The angular separation between groups of maxima and minima is governed by the rotational symmetry of order six (hexagonal) and by the fact that the photonic crystal fiber presents lower resistance to compression in the radial directions of the cross-sectional microstructure with a greater number of aligned holes.

For the photonic crystal fiber and the press rod used in the experiment, we found that the signal intensity change against localized compression force decays exponentially for localized compression forces greater than 400 N for all fixed angular positions of the press used in the experiment.

The parameterized numerical solutions obtained from our theoretical model based on the elasto-optic theory were adjusted to the experimental data considering the physical meaning of each parameter. Thus, the numerical values of each parameter were fixed. The numerical solutions presented the same qualitative behavior as the experimental results and were adjusted to them with a root-mean-square error of 0.0002 for the relative intensity.

In this way, we can explore the cross-section microstructure of a photonic crystal fiber from experimental data obtained with a mechanical compression test and the measurement of the transmitted signal intensity.

**Acknowledgements** We thank Estela Margarita Puente Leos for all her help and willingness as an academic technician at the Acoustics Laboratory of the Department of Physics, Faculty of Sciences, Universidad Nacional Autónoma de México.

In memory of our dear colleague and friend Andrés Valentín Porta Contreras

**Author contributions** All authors contributed to the study conception and design. Data collection and analysis were performed by Alejandro Cortés and Alejandro Sánchez. The first draft of the manuscript was written by Alejandro Sánchez and all authors commented on previous versions of the manuscript. All authors read and approved the final manuscript, and agreed on the order of appearance of the authors.

**Code Availability** Not applicable.

## Declarations

**Competing interests** The authors declare no competing interests.

**Ethics Approval** Not applicable.

**Consent to Participate** Not applicable.

**Consent for Publication** Not applicable.

**Conflict of interest/Competing interests** The authors have no relevant financial or non-financial interests to disclose.

**Open Access** This article is licensed under a Creative Commons Attribution 4.0 International License, which permits use, sharing, adaptation, distribution and reproduction in any medium or format, as long as you give appropriate credit to the original author(s) and the source, provide a link to the Creative Commons licence, and indicate if changes were made. The images or other third party material in this article are included in the article's Creative Commons licence, unless indicated otherwise in a credit line to the material. If material is not included in the article's Creative Commons licence and your intended use is not permitted by statutory regulation or exceeds the permitted use, you will need to obtain permission directly from the copyright holder. To view a copy of this licence, visit http://creativecommons.org/licenses/by/4.0/.

## Data Availability

The data that support the findings of this study are available from the corresponding author upon reasonable request.
